# Dynamic S-acylation of the ER-resident protein stromal interaction molecule 1 (STIM1) is required for store-operated Ca^2+^ entry

**DOI:** 10.1016/j.jbc.2022.102303

**Published:** 2022-08-04

**Authors:** Goutham Kodakandla, Savannah J. West, Qiaochu Wang, Ritika Tewari, Michael X. Zhu, Askar M. Akimzhanov, Darren Boehning

**Affiliations:** 1 Department of Biomedical Sciences, Cooper Medical School of Rowan University, Camden, New Jersey, USA; 2 Department of Biochemistry and Molecular Biology, McGovern Medical School, University of Texas Health Science Center at Houston, Houston, Texas, USA; 3 Department of Integrative Biology and Pharmacology, McGovern Medical School, University of Texas Health Science Center at Houston, Houston, Texas, USA

**Keywords:** Orai1, STIM1, calcium, S-acylation, T cell, store-operated calcium entry, acyl-RAC, acyl resin–assisted capture, BAPTA, 1,2-bis(2-aminophenoxy)ethane-*N*,*N*,*N*′,*N*′-tetraacetic acid, CRAC, calcium release–activated calcium, DHHC, Asp-His-His-Cys, DKO, double knockout, ER, endoplasmic reticulum, HA, hydroxylamine, HEK293, human embryonic kidney 293 cell line, mRFP, monomeric red fluorescent protein, PIP_2_, phosphatidylinositol 4,5-bisphosphate, PLC-γ1, phospholipase C-γ1, PM, plasma membrane, SOAR, STIM1–ORAI activating region, SOCE, store-operated calcium entry, STIM1, stromal interaction molecule 1, TCR, T-cell receptor, TG, thapsigargin, TIRF, total internal reflection fluorescence

## Abstract

Many cell surface stimuli cause calcium release from endoplasmic reticulum (ER) stores to regulate cellular physiology. Upon ER calcium store depletion, the ER-resident protein stromal interaction molecule 1 (STIM1) physically interacts with plasma membrane protein Orai1 to induce calcium release–activated calcium (CRAC) currents that conduct calcium influx from the extracellular milieu. Although the physiological relevance of this process is well established, the mechanism supporting the assembly of these proteins is incompletely understood. Earlier we demonstrated a previously unknown post-translational modification of Orai1 with long-chain fatty acids, known as S-acylation. We found that S-acylation of Orai1 is dynamically regulated in a stimulus-dependent manner and essential for its function as a calcium channel. Here using the acyl resin–assisted capture assay, we show that STIM1 is also rapidly S-acylated at cysteine 437 upon ER calcium store depletion. Using a combination of live cell imaging and electrophysiology approaches with a mutant STIM1 protein, which could not be S-acylated, we determined that the S-acylation of STIM1 is required for the assembly of STIM1 into puncta with Orai1 and full CRAC channel function. Together with the S-acylation of Orai1, our data suggest that stimulus-dependent S-acylation of CRAC channel components Orai1 and STIM1 is a critical mechanism facilitating the CRAC channel assembly and function.

Calcium depletion in the endoplasmic reticulum (ER) upon IP_3_-induced calcium release leads to a rapid, highly controlled, and concerted convergence of plasma membrane (PM) calcium channel Orai1 and ER membrane calcium-sensing protein stromal interaction molecule 1 (STIM1) to form calcium release–activated calcium (CRAC) channel puncta to promote calcium entry (1). The term puncta is used to define these complexes of Orai1 and STIM1 at the ER–PM junctions (2). Calcium entry through these puncta after store depletion is called store-operated calcium entry (SOCE). The luminal EF-hand domain of STIM1 prevents its spontaneous activation by binding to calcium and keeping STIM1 in an inactive conformation (3, 4). Dissociation of calcium from the EF-hand of STIM1 after store depletion results in a conformational change that exposes its STIM1–ORAI activating region (SOAR) (5). STIM1 then oligomerizes and translocates to PM subdomains, where it binds to Orai1 (6, 7). Subsequent calcium entry through Orai1 channels is essential for shaping the spatiotemporal aspects of calcium signaling leading to diverse cellular outcomes.

The mechanism that directs Orai1 and STIM1 into CRAC puncta is still incompletely understood. The prevailing hypothesis is a diffusion trap model, which postulates that a STIM1 conformational change leads STIM1 binding to Orai1 in a stochastic manner at ER–PM contact sites to promote SOCE (8, 9). STIM1 can bind phosphatidylinositol 4,5-bisphosphate (PIP_2_) *via* a lysine-rich C-terminal domain, and studies have shown that STIM1 translocates and binds PIP_2_ present specifically in lipid rafts (10). This indicates an active mechanism for targeting STIM1 to lipid rafts, as we and others have shown previously for Orai1 (11, 12). Perhaps not surprisingly, it has also been shown that there is reduced mobility of Orai1 and STIM1 after store depletion at ER–PM contact sites (13, 14). However, both proteins are in a state of dynamic equilibrium, and both proteins can “escape” a puncta and join other puncta (8). Put together, the assembly of Orai1 and STIM1 at ER–PM junctions is likely more complicated than a simple diffusion trap model with several factors regulating the assembly and disassembly of the CRAC complex.

S-acylation is a reversible post-translational modification of cysteine residues that is mediated by a specific set of enzymes called palmitoyl acyltransferases (15). All known palmitoyl acyltransferases belong to the family of DHHC enzymes owing their name to the aspartate-histidine-histidine-cysteine motif in their catalytic site. S-acylation is known to affect protein stability, trafficking, and recruitment to membrane subdomains (15, 16, 17). In contrast to other post-translational lipid modifications like prenylation, myristoylation, or others, S-acylation is reversible and highly labile (15). We have recently shown that Orai1 is dynamically S-acylated after store depletion and during the initial stages of T-cell activation (11). This was required for Orai1 activation and recruitment into puncta. Here, we show that the dynamic S-acylation of STIM1 also mediates SOCE. STIM1 is rapidly S-acylated after store depletion at cysteine 437, and this is required for recruitment into puncta and SOCE. Our results are consistent with the central role of lipid rafts in mediating the macromolecular assembly of the T-cell receptor (TCR) and other signaling complexes to orchestrate cellular calcium signaling (18).

## Results

### STIM1 is dynamically S-acylated after store depletion at cysteine 437

**Figure 1:**
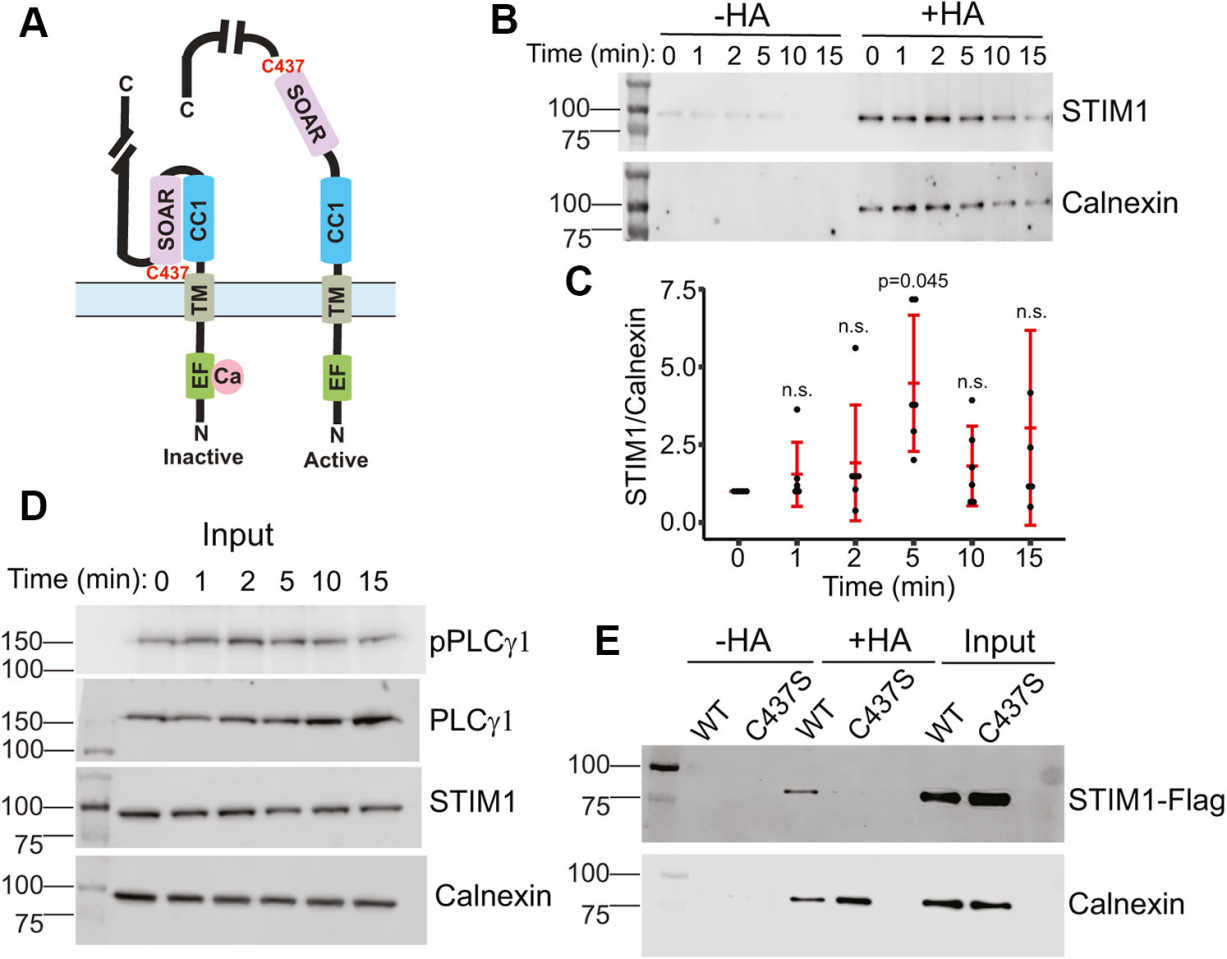
**STIM1 is dynamically S-acylated at cysteine 437.***A*, schematic representation of the inactive and active (extended) conformation of STIM1. The locations of the EF hand, coiled-coil 1 (CC1), and STIM1 Orai1 activation region (SOAR) of STIM1 are indicated. The location of cysteine 437 is noted at the C terminus of the SOAR domain. For simplicity, one monomer of the dimeric STIM1 structure is presented. *B*, Jurkat T cells were treated with anti-CD3 antibody for 0, 1, 2, 5, 10, and 15 min and subjected to acyl-RAC. The reaction without hydroxylamine (−HA) serves as a negative control. Calnexin serves both as a positive control for S-acylation and a loading control. Representative of n = 5. *C*, quantification of fold change of S-acylation of STIM1 normalized to calnexin. Error bars indicate SD. One-way ANOVA showed statistical significance in the level of S-acylation of STIM1 over time (*p* = 0.0372). This was followed up with a Bonferroni post hoc analysis to determine specific time points that showed significance. As presented in Table S1, a significant (*p* = 0.045) increase in S-acylation of STIM1 was observed after 5 min of CD3 addition compared with time 0. *D*, input blots are shown for levels of pPLCγ1, PLCγ1, STIM1, and calnexin. The pPLCγ1 blot is indicative of the time course of TCR activation. *E*, HEK STIM double knockout (DKO) cells were transfected with WT or C437S STIM1-FLAG, and acyl-RAC was performed after 5 min of calcium store depletion with thapsigargin. The blots are representative of four independent experiments. acyl-RAC, acyl resin–assisted capture; HEK, human embryonic kidney cell line; PLCγ1, phospholipase C-γ1; pPLCγ1, phosphorylated phospholipase C-γ1; STIM1, stromal interaction molecule 1; TCR, T-cell receptor.

Previously, we found that S-acylation of Orai1 is increased after stimulation of the TCR in Jurkat T cells (11). We tested the hypothesis that STIM1 also undergoes S-acylation after T-cell activation. STIM1 has only one cytosolic cysteine residue (C437) that can potentially be a substrate for DHHC enzymes (Fig. 1*A*). We treated Jurkat T cells with anti-CD3 (OKT3) antibody to stimulate the TCR in these cells. We obtained cell lysates after different time points and selectively enriched the S-acylated proteins using the acyl resin–assisted capture (acyl-RAC) assay (19). We found that STIM1 is S-acylated after TCR stimulation, peaking at 5 min (Fig. 1, *B* and *C*). These kinetics are consistent with the activation of the downstream TCR signaling cascade indicated by phosphorylation of phospholipase C-γ1 (PLC-γ1) (Fig. 1*D*). Next, to show that C437 is the S-acylated residue in STIM1, we transfected human embryonic kidney 293 (HEK293) STIM1/2 double knockout (DKO) cells with WT Orai1-Myc and WT or C437S versions of STIM1-FLAG. ER calcium store depletion was induced using thapsigargin (TG), and acyl-RAC was performed. Using this approach, we show that WT STIM1, but not the C437S STIM1, is S-acylated after ER calcium store depletion (Fig. 1*E*). Importantly, the expression levels and ER localization of STIM1 were not compromised by the C437S mutant (Fig. S1). Thus, our data suggest that STIM1 is S-acylated at C437 in a stimulus-dependent manner.

### S-acylation of STIM1 at cysteine 437 is required for SOCE

**Figure 2:**
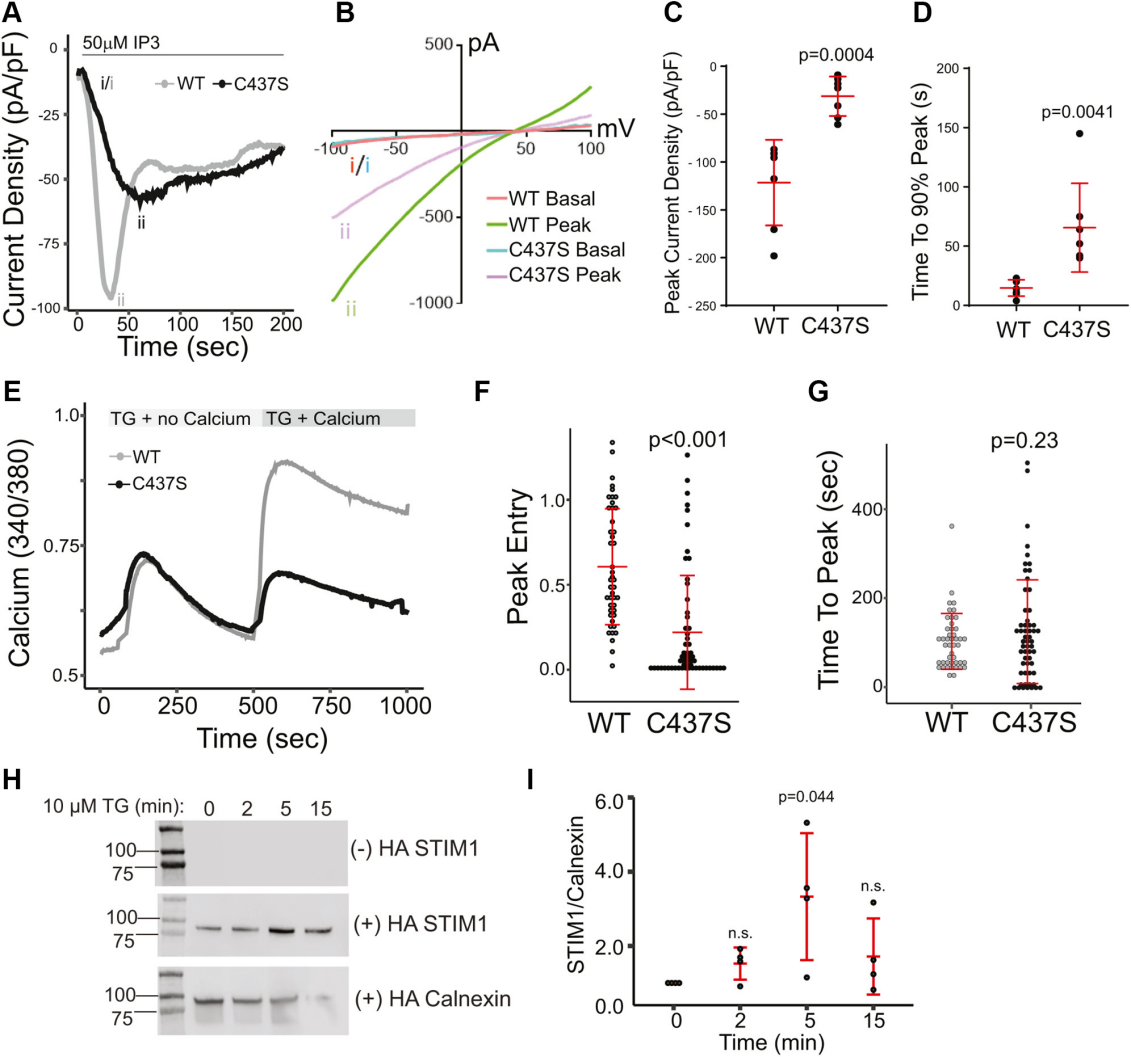
**S-acylation of STIM1 facilitates store-operated calcium entry.***A*, representative traces of CRAC channel currents in cells expressing WT STIM1-mRFP (*gray*) and the acylation-deficient STIM1 mutant C473S (*black*). CRAC channel currents (representative of seven experiments for each condition) were collected at −100 mV immediately upon entering whole-cell configuration. About 50 μM IP3 was included in the pipette to activate IP3 receptors to induce ER Ca^2+^ store depletion. All panels in this figure include coexpression of WT Orai-YFP. *B*, representative current–voltage (I–V) relationships for cells expressing WT or C437S STIM1-mRFP. Labeling on the *left* corresponds to the time point that the I–V curve was collected on the trace in *A*. *C*, quantification of average peak current density of WT and C437S STIM1-mRFP–expressing cells (n = 7 cells for each condition). Error bars indicate SD. *D*, time to 90% peak current in cells expressing WT and C437S STIM1-mRFP. *E*, Fura-2 experiments were conducted in STIM double knockout (DKO) HEK293 cells expressing WT or C437S STIM1-mRFP. Cells were first imaged in Ca^2+^-free medium and then treated with 10 μM thapsigargin (TG) in the absence of Ca^2+^ to induce Ca^2+^ store depletion. Cells were then incubated with 10 μM TG in the presence of 1 mM Ca^2+^ to induce Ca^2+^ entry. Single-cell traces are shown as representatives of three individual experiments. There was no difference in basal Ca^2+^ or peak TG-induced Ca^2+^ release in the two conditions. *F*, quantification of peak Ca^2+^ entry in cells expressing WT or C437S STIM1-mRFP. *G*, quantification of the time to peak Ca^2+^ entry in cells expressing WT and C437S STIM1. In *E* and *F*, each data point represents a single cell, and the data are pooled from three experiments. *H*, HEK293 cells were treated with 10 μM TG for 0, 2, 5, and 15 min and subjected to acyl-RAC. The reaction without hydroxylamine (−HA) serves as a negative control. Calnexin serves both as a positive control for S-acylation and a loading control. *I*, quantification of fold change of S-acylation of STIM1 normalized to calnexin for four experiments. Error bars indicate SD. A one-way ANOVA showed statistical significance in the level of S-acylation of STIM1 over time (*p* = 0.0378). This was followed up with a Bonferroni post hoc analysis to determine the specific time points that showed significance. As presented in Table S2, a significant (*p* = 0.044) increase in S-acylation of STIM1 was observed after 5 min of TG addition compared with time 0. The *p* values in panels *C*, *D*, *F*, and *G* are indicated above the C437S data and were calculated with unpaired two-tailed *t* test. acyl-RAC, acyl resin–assisted capture; CRAC, calcium release–activated calcium; ER, endoplasmic reticulum; HEK293, human embryonic kidney 293 cell line; mRFP, monomeric red fluorescent protein; STIM1, stromal interaction molecule 1.

To evaluate the effect of S-acylation of STIM1 on CRAC channel function, we monitored whole-cell currents in cells coexpressing Orai1-YFP and either WT or the C437S mutant of STIM1-monomeric red fluorescent protein (mRFP). Patch pipettes included 50 μM IP_3_ to actively deplete internal stores and activate CRAC currents. As shown in Figure 2*A* for current density at −100 mV, cells coexpressing Orai1 and WT STIM1 (*gray trace*) displayed current developments with three distinct phases: activation (0–45 s), inactivation (50–100 s), and a plateau (100–150 s). In comparison, the activation phase was markedly slowed for cells coexpressing Orai1 and C437S STIM1 (*black trace*), and the peak current density was also reduced. The current–voltage relationships obtained from voltage ramps at the beginning of whole-cell breaking-in and at the peak of current development showed an inward rectification and reversal potentials at >+40 mV (Fig. 2*B*), consistent with the features of CRAC channels. Peak current density and the time to 90% peak at −100 mV from multiple cells revealed significant decreases in cells that express C437S STIM1 compared with cells that express WT STIM1 (Fig. 2, *C* and *D*).

To further evaluate the effect of S-acylation of STIM1 on SOCE, we used Fura-2 imaging in STIM DKO cells. As shown by others, KO of STIM1 and STIM2 in these cells resulted in a loss of SOCE upon store depletion (Fig. S2; (20)). We rescued STIM1 expression in these cells with either WT or C437S mutant versions of STIM1-mRFP and coexpressed Orai1-YFP. TG in calcium-free buffer was used to induce passive ER calcium store depletion. Calcium addback was achieved using imaging buffer supplemented with 1 mM calcium. Expression of WT STIM1-mRFP rescued SOCE in STIM DKO cells (Fig. 2*E*). In contrast, cells expressing the C437S version of STIM1-mRFP showed significantly reduced calcium entry (Fig. 2*E*). The peak calcium entry in C437S-expressing cells was significantly lower compared with the WT counterpart (Fig. 2*F*), whereas the time to peak was not affected (Fig. 2*G*). STIM1 S-acylation induced by TG treatment as determined by acyl-RAC was significant at 5 min (Fig. 2,*H* and *I*). This is kinetically consistent with the anti-CD3 data in Jurkat cells (Fig. 1*C*) and the Fura-2 time to peak data (Fig. 2*G*). We conclude that cysteine 437 on STIM1 is required for SOCE.

### S-acylation of STIM1 is required for Orai1–STIM1 assembly

**Figure 3:**
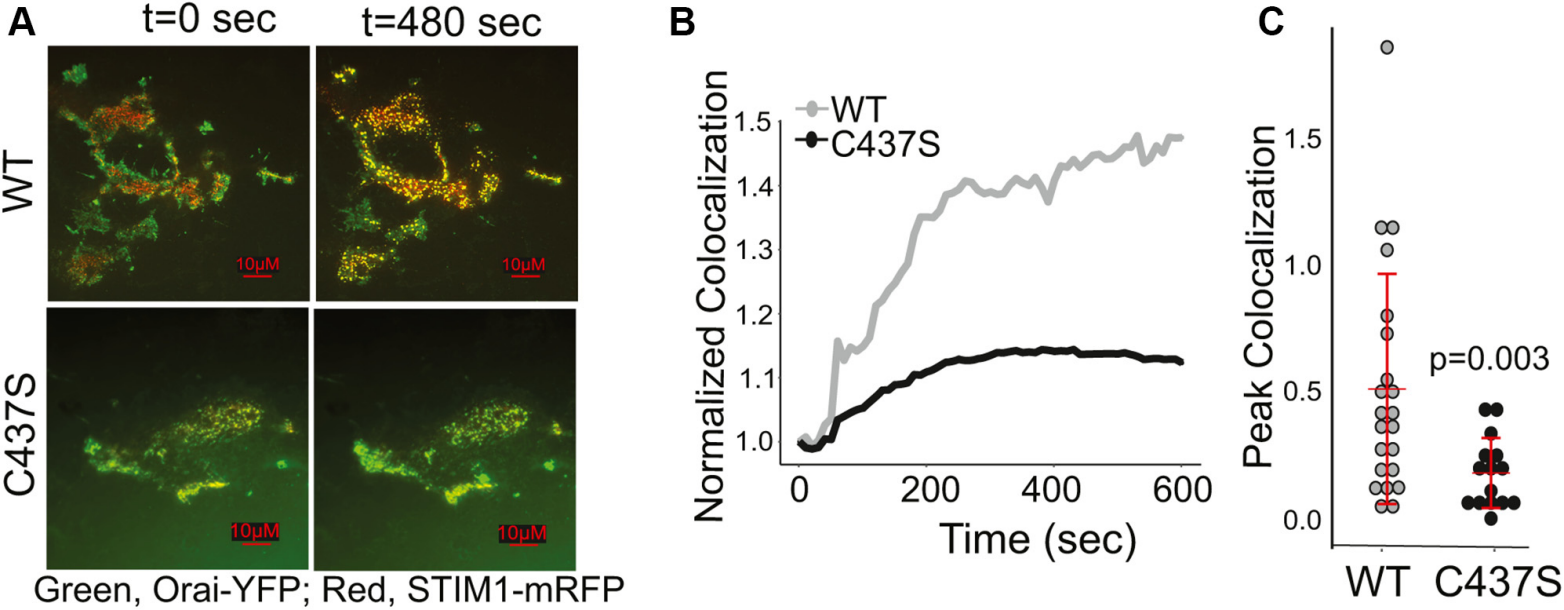
**S-acylation of STIM1 is required for colocalization with Orai1.***A*, representative TIRF images from six experiments of STIM DKO cells transfected with WT Orai1-YFP (*green*) and either WT (*top*) or C437S (*bottom*) STIM1-mRFP (*red*). Thapsigargin was added after 60 s of baseline recording. See also Videos S1 and S2. *B*, quantification of WT and C437S STIM1-mRFP colocalization with Orai1-YFP using Pearson's correlation coefficient over time. *Traces* represent averages pooled from six separate experiments. The data were normalized to time 0. *C*, peak normalized colocalization quantification of WT and C437S STIM1-mRFP and Orai1-YFP. Each data point represents a single cell, and the data are pooled from six separate experiments. Error bars indicate SD. The *p* value was calculated with an unpaired two-tailed *t* test. DKO, double knockout; mRFP, monomeric red fluorescent protein; STIM1, stromal interaction molecule 1; TIRF, total internal reflection fluorescence.

We previously showed that preventing S-acylation of Orai1 results in loss of puncta formation and colocalization with STIM1 upon store depletion (11). We next tested whether S-acylation of STIM1 is required for assembly with Orai1. We cotransfected STIM DKO cells with Orai1-YFP and either WT or C437S versions of STIM1-mRFP and depleted ER stores using 10 μM TG. Cells cotransfected with Orai1-YFP and WT STIM1-mRFP showed a rapid and stimulus-dependent colocalization by total internal reflection fluorescence (TIRF) microscopy imaging (Figs. 3, *A*–*C*, and S3). In contrast, cells expressing the C437S version of STIM1 showed slower kinetics of colocalization with Orai1 and significantly reduced peak colocalization (Figs. 3, *A*–*C*, and S3).

### Formation of functional Orai1–STIM1 puncta is compromised by the STIM1 C437S mutant

**Figure 4:**
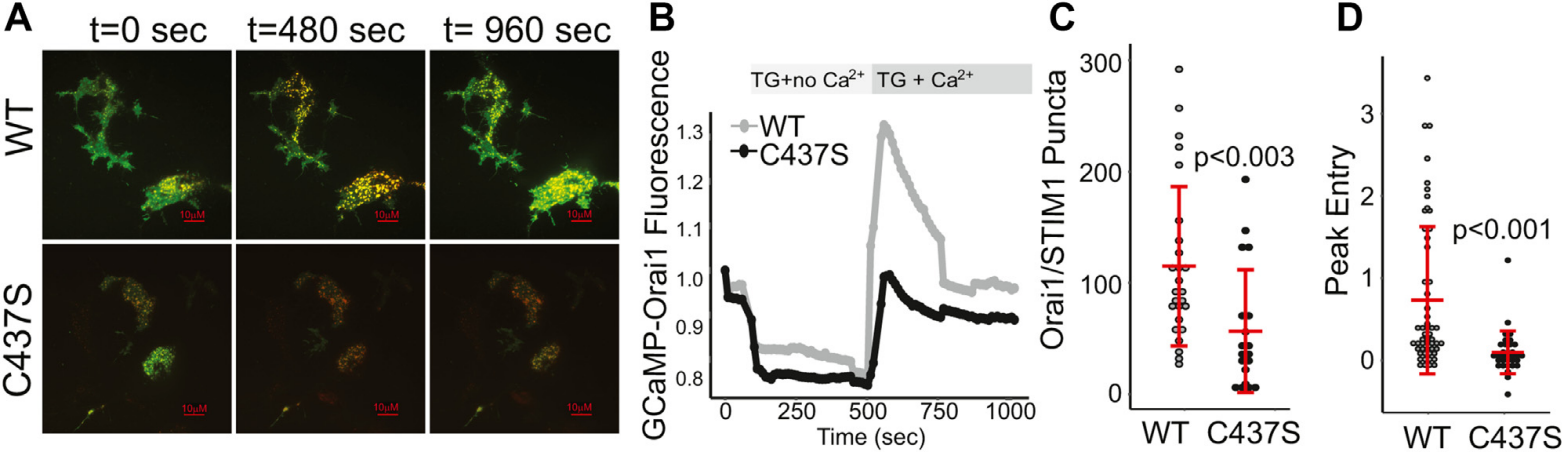
**STIM1 S-acylation is required for the recruitment of active Orai1 to puncta.***A*, representative TIRF images from six experiments of STIM DKO cells transfected with WT Orai-GCaMP6f (*green*) and either WT (*top*) or C437S (*bottom*) STIM1-mRFP (*red*). Cells were first imaged in Ca^2+^-free medium and then treated with 10 μM thapsigargin (TG) in the absence of Ca^2+^ to induce Ca^2+^ store depletion. Cells were then incubated with 10 μM TG in the presence of 1 mM Ca^2+^ to induce Ca^2+^ entry. See Videos S3 and S4. *B*, normalized Orai-GCaMP6f fluorescence of cells transfected with WT and C437S STIM1-mRFP over time. *Traces* represent averages pooled from six separate experiments. *C*, quantification of the number of Orai1–GCaMP6f/STIM1 puncta, which appear after Ca^2+^ addback. *D*, peak normalized fluorescence of Orai1-GCaMP6f in cells expressing WT and C437S STIM1-mRFP. Each data point in panels *C* and *D* represents a single cell, and the data are pooled from six separate experiments. Error bars indicate SD. The *p* values were calculated using an unpaired two-tailed *t* test. DKO, double knockout; mRFP, monomeric red fluorescent protein; STIM1, stromal interaction molecule 1; TIRF, total internal reflection fluorescence.

Orai1 fused to the calcium indicator GCaMP6f allows the direct and local imaging of calcium entry through individual or clusters of Orai1 channels by TIRF imaging (21). We have previously used this approach to investigate how S-acylation of Orai1 affects the formation of active Orai1 puncta (11). To investigate how STIM1 S-acylation affects the Orai1 channel activation and puncta formation, we cotransfected STIM DKO cells with WT Orai1-GCaMP6f and either the WT or C437S version of STIM1-mRFP. The addition of 10 μM TG in calcium-free media led to puncta formation in WT STIM1-expressing cells, with much fewer puncta in C437S-expressing cells (Figs. 4, *A*–*C*, and S4). TG treatment in calcium-free media resulted in no increase in Orai1-GCaMP6f fluorescence in either WT or C437S STIM1-expressing cells, consistent with the specificity of this protein in measuring calcium entry through the mouth of the channel (Figs. 4, *A*, *B*, and S4). Calcium addback caused a significant increase in Orai1-GCaMP6f fluorescence in cells expressing WT STIM1, indicative of calcium entry through Orai1 channels. In contrast, cells expressing C437S STIM1 did not show a significant increase in fluorescence after calcium addback but only recovered to the level comparable to that of before extracellular calcium removal (Figs. 4, *A*, *B*, *D*, and S4). We conclude that the STIM1-C437S mutant has defects in both the recruitment to Orai1 puncta and gating the channel.

## Discussion

SOCE is an important mechanism for calcium refilling after ER calcium store depletion and contributes significantly to shaping the spatiotemporal aspects of calcium signaling (22, 23). The mechanisms behind how the CRAC channel components are translocated to the ER–PM junctions to form puncta are not fully understood. Previously, our group demonstrated that Orai1 is dynamically S-acylated after store depletion, and a cysteine mutant version of this protein that cannot undergo S-acylation affects CRAC channel assembly and SOCE (11), findings that have also been validated by others (12). Here, we show that S-acylation of STIM1 also plays a crucial role in CRAC puncta formation and that it contributes to SOCE.

**Figure 5:**
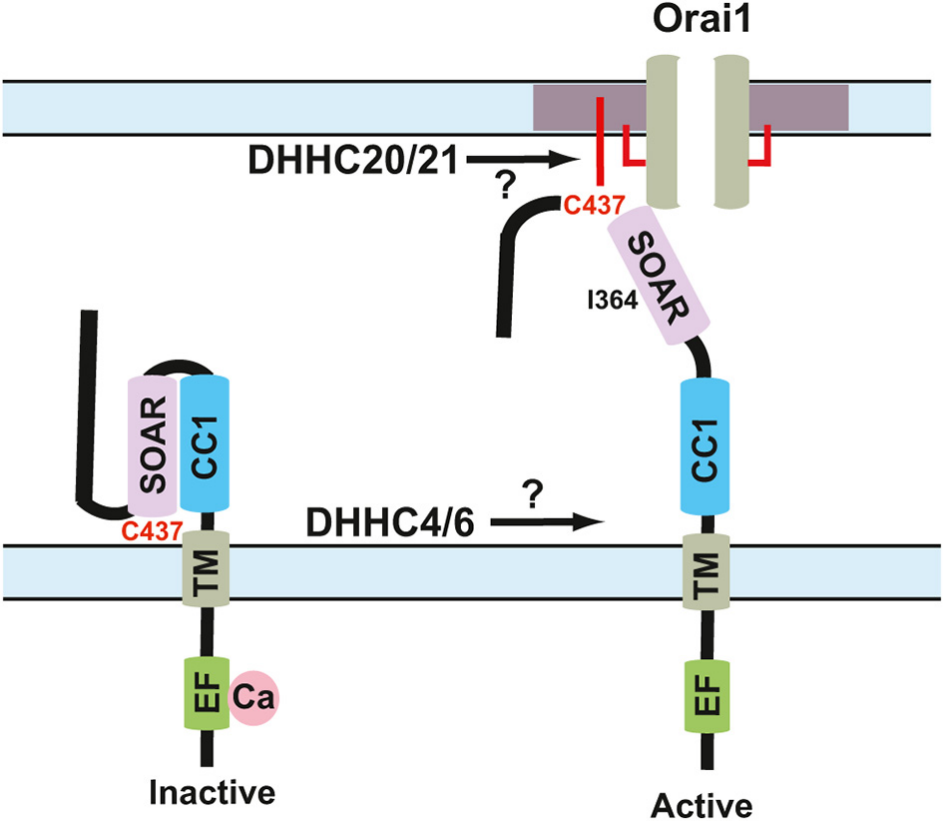
**S-acylation of STIM1 C437 and CRAC channel function.** Two potential models for the regulation of STIM1 by S-acylation at C437. In the first model, ER-localized DHHC4/6 S-acylates C437 facilitating and/or stabilizing the extended conformation of STIM1. In the second model, S-acylation of C437 by the plasma membrane–localized DHHC20/21 both stabilizes the extended conformation and promotes recruitment to lipid rafts, where it can bind to and gate S-acylated Orai1. The cholesterol-binding residue isoleucine 364 is also highlighted. For clarity, the C-terminal PIP_2_-binding polybasic domain is not shown. CRAC, calcium release–activated calcium; DHHC, Asp-His-His-Cys; ER, endoplasmic reticulum; PIP_2_, phosphatidylinositol 4,5-bisphosphate; STIM1, stromal interaction molecule 1.

The S-acylation of Orai1 in the PM targets the channel to lipid rafts where it forms CRAC channels with STIM1 to promote calcium entry (11, 12). We hypothesize that S-acylation of STIM1 regulates SOCE in two possible ways. One model postulates that STIM1 is S-acylated by an ER-localized DHHC enzyme to promote and/or stabilize the extended conformation of STIM1 (Fig. 5). Another possible model hypothesizes the S-acylation of the C terminus of STIM1 in the extended conformation by a PM-localized DHHC enzyme. In this model, S-acylation would function not only to anchor the SOAR–CRAC activation domain of STIM1 in the PM but also promote localization to lipid rafts to facilitate binding to raft-localized Orai1 (Fig. 5). Importantly, this model proposes that both the assembly and disassembly of CRAC channels is an enzymatically regulated process. If this model is correct, a significant revision to the diffusion-trap model of CRAC channel assembly would be required. The persistence of significant SOCE in STIM1-C437S expressing cells suggests that S-acylation cannot be the only enzymatic mechanism driving Orai–STIM assembly and disassembly. Future work will likely identify a multifaceted mechanism for the regulation of SOCE and the potential role of DHHC enzymes in this process.

Active STIM1 adopts an extended conformation upon store depletion. This fully extended conformation facilitates binding of the polybasic domain of STIM1 to the PIP_2_-rich domains specifically in PM lipid rafts (10). In addition, STIM1 has a cholesterol-binding domain in the SOAR region, of which isoleucine 364 plays a critical role (24). Thus, in addition to S-acylation, there are multiple structural features in the C terminus of STIM1, which promotes binding and segregation to lipid rafts. This redundancy in function is a likely explanation for the partial loss of SOCE we describe here with the C437S mutant STIM1. Interestingly, it has been shown that STIM1 encompassing only residues 1 to 442 still forms puncta in Orai1 triple KO cells, whereas STIM1 encompassing residues 1 to 342 does not (25). Finally, we show that STIM1 can form puncta in Orai triple KO cells upon store depletion using TG (Fig. S5). These results indicate that Orai1 and the polybasic domain of STIM1 are dispensable for puncta formation; however, the SOAR domain (containing C437 and I364 residues) is absolutely required. It has previously been shown that a C437G STIM1 mutant has reduced calcium entry similar to our results (26); however, this mutant retained the ability to form puncta. As these experiments were performed in WT HEK cells, we conclude that the C437G mutant can associate with endogenous STIM1 to rescue puncta formation.

It is possible that the same PM DHHC enzyme mediates the S-acylation of both Orai1 and STIM1. This hypothesis is supported by our findings that both Orai1 and STIM1 reach peak S-acylation levels at the same time point after T-cell stimulation (Fig. 1, *B* and *C*; (11)). Similarly, we have observed in TIRF time lapse imaging experiments that the peak colocalization of Orai1 and STIM1 happens approximately at the same time after store depletion (Fig. 3*B*; (11)). It has been shown that DHHC20 S-acylates Orai1 (12). We have shown that many TCR pathway components are S-acylated by the PM-localized DHHC21. We have shown that the DHHC21 binds to calcium/calmodulin (27). Mutation of the calcium/calmodulin site of DHHC21 in the *depilated* mouse leads to defects in T-cell activation and differentiation into its subsets establishing *in vivo* relevance (27, 28, 29, 30). Future work will evaluate if Orai1 can also be S-acylated by DHHC21 and determine the DHHC enzyme(s) responsible for STIM1 S-acylation. Of note, the dramatic defects in T-cell function in DHHC21 mutant mice may be consistent with alterations in SOCE (27).

It is possible that cysteine 437 in STIM1 can be susceptible to other post-translational modifications. For example, the Orai–STIM complex can be regulated by the oxidation of cysteine residues. STIM2 shares the homologous cysteine at position 437 but has an additional 10 cytosolic cysteine residues. It has been shown that only cysteine 313 is subject to oxidation in STIM2, suggesting that STIM1 is not subject to redox regulation at C437 (31). Other cysteine modifications such as S-nitrosylation are also a possibility; however, our results clearly show that dynamic S-acylation is prominent at C437.

We can now include the Orai1–STIM1 complex in the dynamically S-acylated proteome of T cells. As such, CRAC components are colocalized to the immunological synapse with TCR signaling proteins following cellular stimulation to promote sustained calcium entry that is required for immune responses (1, 32). Our results also indicate that the S-acylation enzymatic machinery may be a viable therapeutic target for diseases of immune cell function.

## Experimental procedures

### Cells, antibodies, and constructs

HEK293 STIM1/2 DKO cells were a kind gift of Dr Mohamed Trebak (20) (University of Pittsburgh) and were maintained in Dulbecco’s modified Eagle’s medium supplemented with 10% fetal bovine serum, 1% l-glutamine, and 1% penicillin–streptomycin. Jurkat T cells were obtained from American Type Culture Collection and cultured in RPMI media supplemented with 10% fetal bovine serum, 1% l-glutamine, and 1% penicillin–streptomycin. Cells were plated on polystyrene tissue culture dishes or 6-well plates for experiments. For some experiments, cells were plated on poly-l-lysine–coated coverslips for imaging. Cells were transfected with Lipofectamine 3000 (Invitrogen) according to the manufacturer’s protocol with 0.75 μg Orai1 plasmid and/or 2.25 μg STIM1 plasmid per 35-mm well. Cells were maintained at 37 °C and 5% CO_2_. For immunoblotting, the antibodies were purchased from commercially available sources: STIM1 (catalog no.: 4961S), calnexin (catalog no.: 2679), PLC-γ1 (catalog no.: 2822S), phospho-PLC-γ1 (Tyr783) (catalog no.: 2821S), anti-rabbit immunoglobulin, horseradish peroxidase–linked secondary antibody (catalog no.: 7074S), antimouse immunoglobulin, and horseradish peroxidase–linked secondary antibody (catalog no.: 7076P2) were from Cell Signaling Technology; anti-CD3 human antibody (catalog no.: 14-0037-82) was from eBiosciences. Orai1-YFP was a generous gift from Anjana Rao (Addgene plasmid #19756). Orai1-GCaMP6f was a plasmid deposited in Addgene by Michael Cahalan (Addgene #73564; (21)). STIM1-mRFP was a generous gift from David Holowka and Barbara Baird (Cornell University). The C437S mutant version of STIM1-mRFP and STIM1-FLAG was prepared using q5 site-directed mutagenesis kit from New England Biolabs. The following primers for mutagenesis were designed using the NEB tool 5′-GAGATCAACCTTGCTAAGCAGG-3′ and 5′-GAAACACACTCTTTGGCACCTT-3′. The mutation was confirmed using Sanger sequencing (Eton Biosciences). Thiol-sepharose beads used for acyl-RAC were obtained from Nanocs, Inc and activated according to the manufacturer’s protocol before continuing with acyl-RAC protocol listed later. All other chemicals and reagents were purchased from Sigma–Aldrich or VWR.

### Electrophysiology

We performed whole-cell recording using HEK293 cells coexpressing Orai1-YFP with WT or C437S STIM1-mRFP as described previously (11). Patch clamping was conducted with an EPC-10 amplifier controlled by PatchMaster software (HEKA) within 24 h after transfection. The resistance of fire polished pipettes was between 5 MΩ and 8 MΩ. The pipette solution contained 120 mM cesium glutamate, 20 mM cesium, 20 mM BAPTA (1,2-bis(2-aminophenoxy)ethane-N,N,N′,N′-tetraacetic acid), 3 mM MgCl_2_, 0.05 mM IP_3_ (K^+^-salt), and 10 mM Hepes (pH 7.2 with CsOH). The bath solution contained 120 mM NaCl, 10 mM CaCl_2_, 2 mM MgCl_2_, 10 mM tetraethylammonium–HCl (tetraethylammonium chloride), 10 mM glucose, and 10 mM Hepes (pH 7.2 with NaOH). Cells were held at the holding potential of 0 mV, and currents were recorded at a sampling frequency of 10 kHz with 1 kHz filtering. Currents were elicited by voltage ramps from −100 to +100 mV in 100 mS, repeated every second. To capture current development immediately after IP_3_ dialysis, the recording began before the establishment of the whole-cell configuration and continued throughout the breaking-in and afterward. Patches with seal resistance up to 3 GΩ to 5 GΩ were broken by applying negative pressure, and only cells that maintained GΩ resistance after the breaking-in were used for analysis. All voltages were corrected for a liquid junction potential of −10 mV. Passive store depletion protocols using BAPTA only or BAPTA plus TG were found to be rate limiting and thus are not appropriate for examining the kinetics of SOCE activation in these experiments.

### Fura-2 imaging

Calcium imaging on HEK293 cells was performed as previously described (33). Briefly, 2 days before imaging, approximately 300,000 HEK293 STIM DKO cells were seeded on coverslips in a 6-well culture plate. After 24 h, these cells were transfected with WT Orai1-YFP and either WT or C437S STIM1-mRFP plasmids. All calcium imagings were performed in 1% bovine serum albumin, 107 mM NaCl, 20 mM Hepes, 2.5 mM MgCl_2_, 7.5 mM KCl, and 11.5 mM glucose with or without the presence of 1 mM CaCl_2_. Time-lapse images were obtained every 2 s for 16 min using a 40× oil immersion objective lens. Cells were excited using 340 and 380 nm wavelengths alternatively every 2 s, and emission was collected at 525 nm. The first minute of recording was used as baseline. After 1 min, 10 μM TG in calcium-free imaging solution was added to induce ER calcium store depletion. This was continued for 7 min, and imaging solution with calcium and TG was added back to induce calcium entry. The ratio of fluorescence at 340 to 380 nm was used to quantify cytosolic calcium. We excluded the cells that did not respond to TG in our analysis. For calculating peak entry, we subtracted the maximum fluorescence ratio (*R*_max_) after calcium addback from the fluorescence ratio at time 0 (*R*_0_) and normalized to *R*_0_.

### Confocal imaging

HEK293 STIM DKO cells were plated on coverslips in a 6-well plate at a density of 350,000 cells per coverslip. The next day, they were transfected with WT or C437S version of STIM1-mRFP using Lipofectamine 3000 per the manufacturer’s protocol. The next day, cells were imaged using Nikon Ti2 confocal microscope with a plan apo lambda 60× oil objective at 1.5 magnification factor. Exposure parameters were identical between the WT- and STIM1-expressing cells.

### Acyl-RAC assay

We performed the acyl-RAC assay as described by our group elsewhere (19). Cells were transfected with WT Orai1-Myc and either WT or C437S version of STIM1-FLAG plasmids. For Jurkat T cells, 10 million cells per treatment were used for endogenous STIM1 S-acylation experiments and were treated with 5 μg/ml anti-CD3 (OKT3) antibody. For STIM1 exogenous expression paradigms, we used 10 μM TG for 5 min to induce ER calcium store depletion. Cell lysates were collected after the specific time points using 1% dodecyl ß-d-maltoside in PBS, supplemented with cOmplete protease inhibitor cocktail (Roche), acyl protein thioesterase inhibitor ML211, and serine protease inhibitor PMSF (10 mM). The lysates were cleared at 20,000*g* for 30 min at 4 °C by centrifugation. Approximately 500 μg of cell lysates were used for the assay. The lysates were precipitated using 2:1 methanol and chloroform, and the resulting protein pellets were incubated with 0.2% methylmethanethiosulfonate for 15 min at 42 °C. Methylmethanethiosulfonate was removed using three rounds of methanol chloroform precipitation. After the last round of precipitation, the pellets were dissolved in 2SHB buffer (2% SDS, 5 mM EDTA, 100 mM Hepes, and pH 7.4). About 20 μl of lysate was saved for input. The lysates were incubated with 400 mM hydroxylamine (HA) to cleave the thioester bonds and incubated with thiolsepharose resin overnight at 4 °C. For the minus HA controls, lysates were incubated with 400 mM sodium chloride instead of HA. The next day, the samples were washed four times using 1% SDS solution in buffer A (5 mM EDTA, 100 mM Hepes, and pH 7.4) and eluted using 10 mM DTT in SDS buffer (1% SDS, 50 mM Tris–HCl, 10% glycerol, and 1% bromophenol blue). The samples were resolved on 10% SDS-PAGE gels and analyzed using Western blotting.

### TIRF

HEK293 STIM DKO cells were seeded on coverslips treated with poly-l-lysine 2 days before imaging at 300,000 cells per coverslip. The next day, cells were transfected with WT Orai1-YFP or either WT or C437S STIM1-mRFP. For TIRF imaging, we used a Nikon Eclipse Ti microscope equipped with TIRF illumination system and a 60× oil objective. The cells were mounted on the microscope and alternatively excited using 488 and 561 nm lasers every 10 s. The images were acquired for 8 min using a Photometrics Prime 95B camera. The first minute of the recording was used as baseline. TG in calcium-free imaging solution was added after the first minute to observe Orai1 and STIM1 PM targeting and recruitment to CRAC puncta. For the experiments in Figure 4, we used Orai1-GCaMP6f with WT or C437S version of STIM1-mRFP to evaluate the effect of STIM1 S-acylation on Orai1 channel activity. We altered the protocol to evaluate calcium entry and imaged every 5 s. Imaging was done for 16 min, and the first minute was used as baseline. After 1 min, TG in calcium-free imaging solution was added to observe STIM1 colocalization with Orai1. After 8 min, the solution was replaced with imaging solution with calcium to observe calcium entry. We excluded all cells with aggregates under resting conditions from our analyses. We used Nikon NIS Elements software for fluorescence data analysis. We drew regions of interest around cells to calculate relative fluorescence units at any point during the time lapse. We normalized the relative fluorescence unit of specific cell to itself by dividing a specific time point fluorescence (C_n_) value to time 0 (C_0_). We used these normalized fluorescence values to plot Orai1 channel fluorescence over the time course of TG addition and calcium addback. To calculate the peak entry, we obtained the maximum fluorescence value (C_max_) after calcium addback and subtracted it from the fluorescence value at time 0 (C_0_) and normalized the difference by dividing it to time 0 (C_0_) (C_max_ − C_0_/C_0_). To calculate the colocalization between Orai1 and STIM1, we used colocalization analysis on Nikon NIS Elements software. The software uses Pearson’s correlation coefficient as an estimate for colocalization between two fluorophores. We drew regions of interest around cells of interest and obtained correlation coefficients for the time series of all cells. We followed a similar method of normalized correlation by dividing the correlation coefficient at a given time point (R_n_) by time 0 (R_0_). We used these values to plot the colocalization curve. To calculate peak colocalization, we used a similar method to that of peak entry, subtracted the peak colocalization after TG addition (R_max_) from time zero (R_0_), and divided the difference to time 0 (R_0_) ((R_max_ − R_0_)/R_0_).

## Data availability

All data supporting this study are available from the corresponding author (boehning@rowan.edu) upon request.

## Supporting information

This article contains supporting information.

## Conflict of interest

The authors declare that they have no conflicts of interest with the contents of this article.
