# Supplementary material for: Dynamic S-acylation of the ER-resident protein stromal interaction molecule 1 (STIM1) is required for store-operated Ca2+ entry
Source: J Biol Chem. 2022 Aug 4;298(9):102303. doi: 10.1016/j.jbc.2022.102303 (PMC9463532; doi:10.1016/j.jbc.2022.102303)
Supplement: Supplementary Table 1 [file mmc5.docx]

**Supplementary Table 1**

Summary of parameters one-way ANOVA for **Figure 1C**

|  | Degrees of Freedom | Sum or Squares | Mean of Squares | F value | P value |
| --- | --- | --- | --- | --- | --- |
| Time | 5 | 47.51 | 9.502 | 2.742 | 0.0372 |
| Residuals | 30 | 103.95 | 3.465 |  |  |

Pairwise comparisons using t tests with pooled SD

|  | 0 min | 1 min | 2 min | 10 min | 15 min |
| --- | --- | --- | --- | --- | --- |
| 1 min | 1.000 | - | - | - | - |
| 2 min | 1.000 | 1.000 | - | 1.000 | 1.000 |
| 5 min | 0.045 | 0.161 | 0.356 | 0.287 | 1.000 |
| 10 min | 1.000 | 1.000 | - | - | - |
| 15 min | 1.000 | 1.000 | - | 1.000 | - |

Post-hoc adjustment method: Bonferroni correction. Significant values are highlighted.
