# Supplementary material for: Dynamic S-acylation of the ER-resident protein stromal interaction molecule 1 (STIM1) is required for store-operated Ca2+ entry
Source: J Biol Chem. 2022 Aug 4;298(9):102303. doi: 10.1016/j.jbc.2022.102303 (PMC9463532; doi:10.1016/j.jbc.2022.102303)
Supplement: Supplementary Table 2 [file mmc6.docx]

**Supplementary Table 2**

Summary of parameters one-way ANOVA for **Figure 2I**

|  | Degrees of Freedom | Sum or Squares | Mean of Squares | F value | P value |
| --- | --- | --- | --- | --- | --- |
| Time | 3 | 12.09 | 4.031 | 3.873 | 0.0378 |
| Residuals | 12 | 12.49 | 1.041 |  |  |

Pairwise comparisons using t tests with pooled SD

|  | 0min | 2min | 15min |
| --- | --- | --- | --- |
| 2min | 1.0000 | - | 1.0000 |
| 5min | 0.044 | 0.168 | 0.269 |
| 15min | 1.0000 | - | - |

Post-hoc adjustment method: Bonferroni correction. Significant values are highlighted.
