# Supplementary figures and images for: Dynamic S-acylation of the ER-resident protein stromal interaction molecule 1 (STIM1) is required for store-operated Ca2+ entry
Source: J Biol Chem. 2022 Aug 4;298(9):102303. doi: 10.1016/j.jbc.2022.102303 (PMC9463532; doi:10.1016/j.jbc.2022.102303)

A

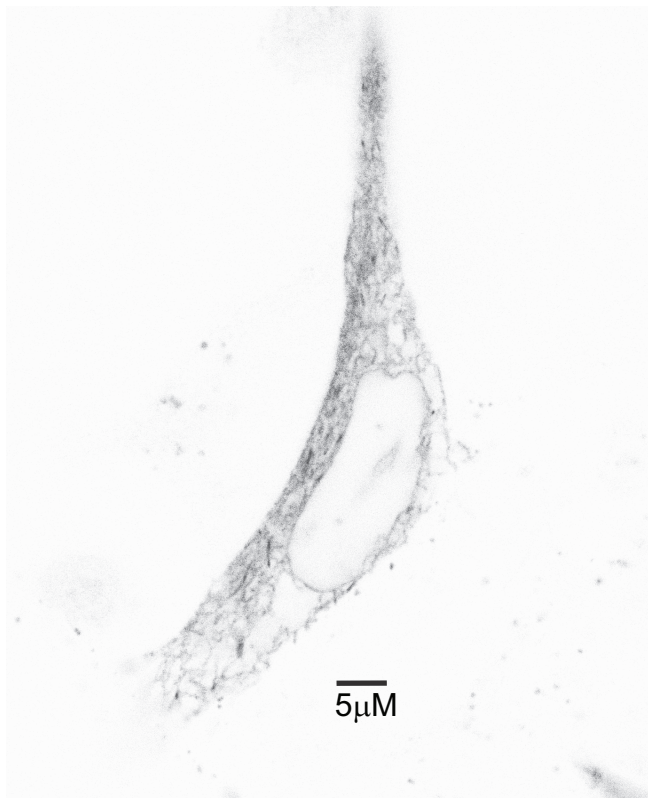

WT

B

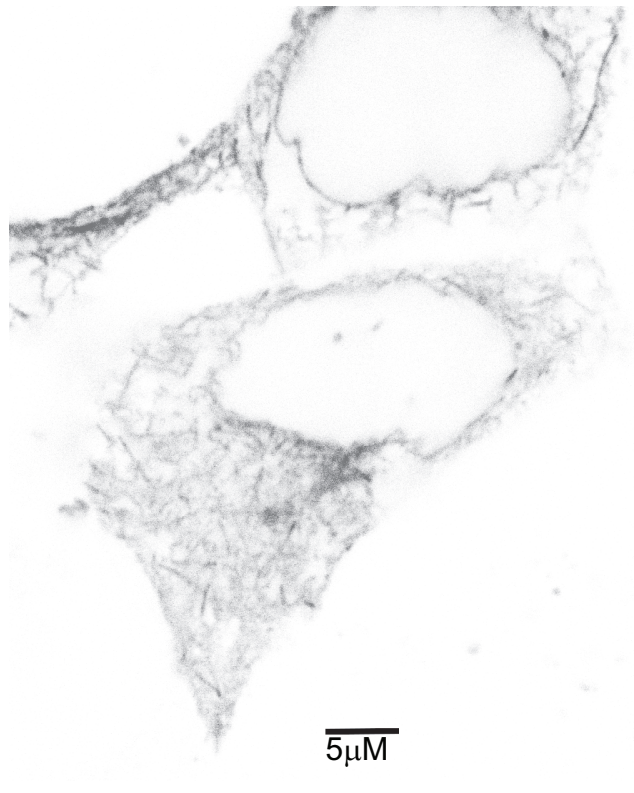

C437S

Supplement: Supplemental Figure 1 [file mmc7.pdf]

A

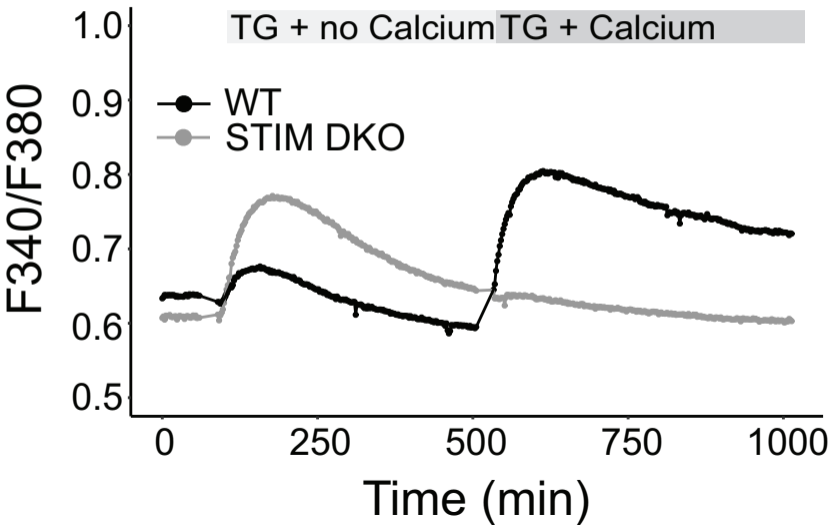

B

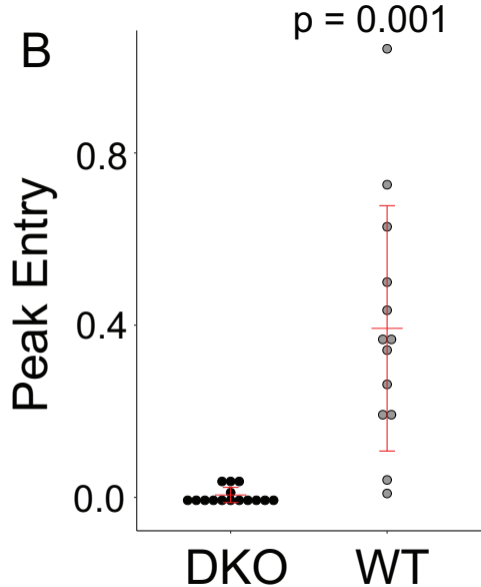

Supplement: Supplemental Figure 2 [file mmc8.pdf]

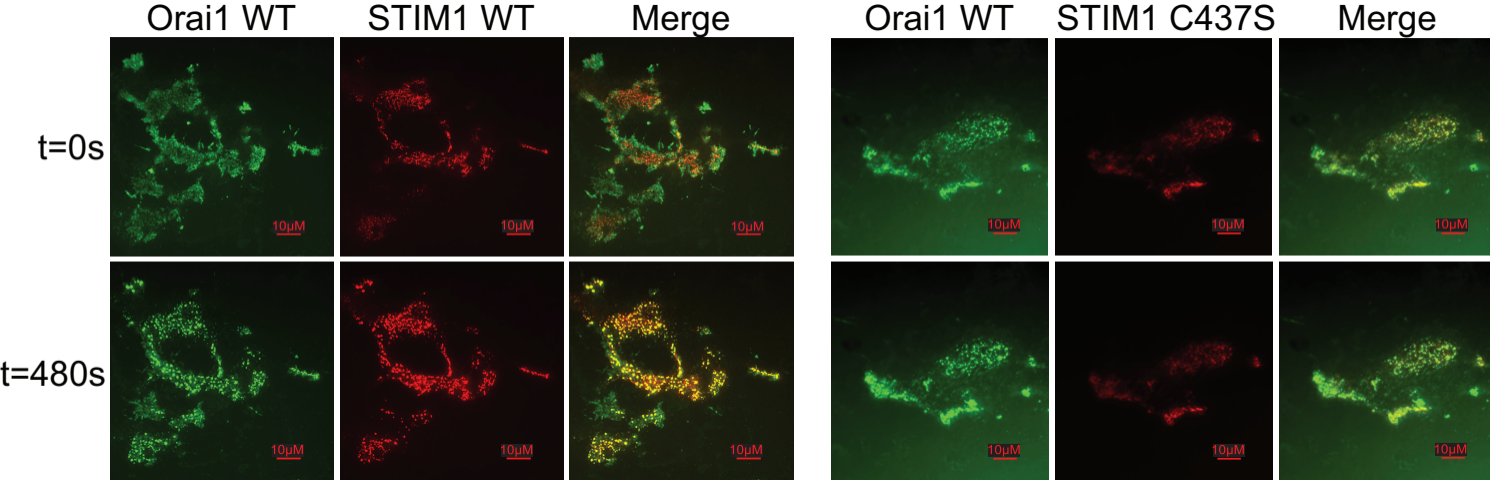

Supplement: Supplemental Figure 3 [file mmc9.pdf]

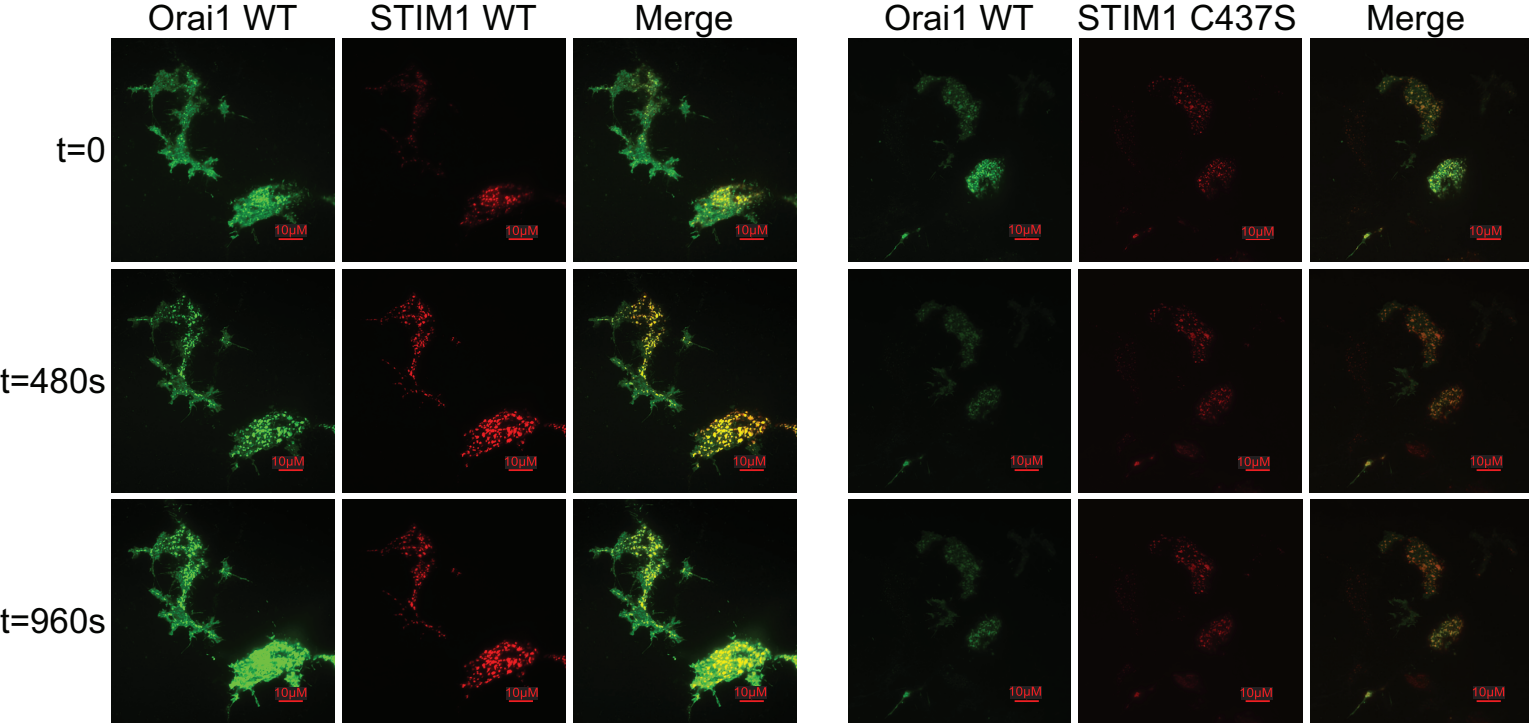

Supplement: Supplemental Figure 4 [file mmc10.pdf]

t=0

t=480s

STIM1 WT

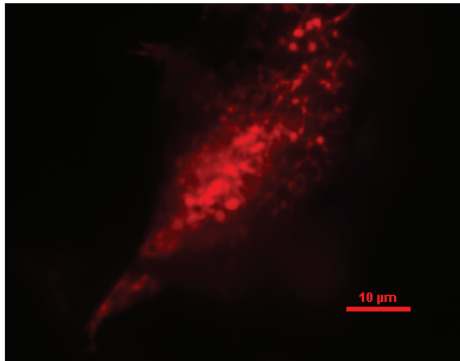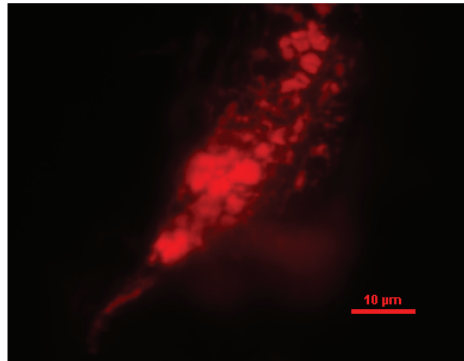

STIM1 C437S

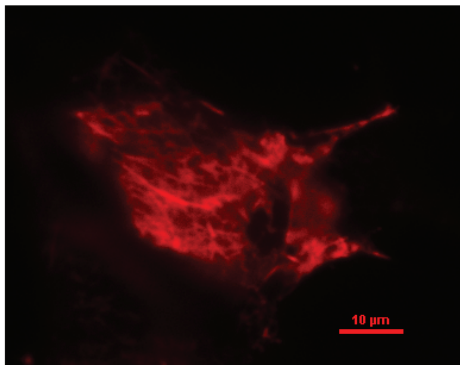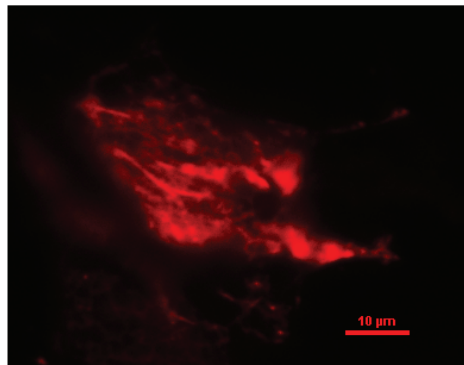

Supplement: Supplemental Figure 5 [file mmc11.pdf]
